# Time Course Based Artifact Identification for Independent Components of Resting-State fMRI

**DOI:** 10.3389/fnhum.2013.00214

**Published:** 2013-05-23

**Authors:** Christian Rummel, Rajeev Kumar Verma, Veronika Schöpf, Eugenio Abela, Martinus Hauf, José Fernando Zapata Berruecos, Roland Wiest

**Affiliations:** ^1^Support Center for Advanced Neuroimaging, University Institute for Diagnostic and Interventional Neuroradiology, Inselspital – Bern University Hospital, University of BernSwitzerland; ^2^Division of Neuro- and Musculoskeletal Radiology, Department of Radiology, Medical University of ViennaVienna, Austria; ^3^Department of Neurology, Inselspital – Bern University Hospital, University of BernSwitzerland; ^4^Klinik Bethesda TschuggBern, Switzerland; ^5^Universidad Pontíficia BolivarianaMedellín, Columbia; ^6^Instituto Neurologico de AntioquiaMedellín, Columbia

**Keywords:** ICA, resting-state networks, fMRI, BOLD, artifacts, group studies

## Abstract

In functional magnetic resonance imaging (fMRI) coherent oscillations of the blood oxygen level-dependent (BOLD) signal can be detected. These arise when brain regions respond to external stimuli or are activated by tasks. The same networks have been characterized during wakeful rest when functional connectivity of the human brain is organized in generic resting-state networks (RSN). Alterations of RSN emerge as neurobiological markers of pathological conditions such as altered mental state. In single-subject fMRI data the coherent components can be identified by blind source separation of the pre-processed BOLD data using spatial independent component analysis (ICA) and related approaches. The resulting maps may represent physiological RSNs or may be due to various artifacts. In this methodological study, we propose a conceptually simple and fully automatic time course based filtering procedure to detect obvious artifacts in the ICA output for resting-state fMRI. The filter is trained on six and tested on 29 healthy subjects, yielding mean filter accuracy, sensitivity and specificity of 0.80, 0.82, and 0.75 in out-of-sample tests. To estimate the impact of clearly artifactual single-subject components on group resting-state studies we analyze unfiltered and filtered output with a second level ICA procedure. Although the automated filter does not reach performance values of visual analysis by human raters, we propose that resting-state compatible analysis of ICA time courses could be very useful to complement the existing map or task/event oriented artifact classification algorithms.

## 1 Introduction

Functional magnetic resonance imaging (fMRI) technologies have nowadays been implemented into various clinical applications, e.g., pre-surgical mapping of eloquent areas of the brain before resective surgery in brain tumors and epilepsy (Gutbrod et al., 2012; Kollndorfer et al., 2013). The basic principle of fMRI lies in the statistical testing of changes in the blood oxygen level-dependent (BOLD) signal induced by either a given task or correlations with endogenous stimuli in the brain, as interictal epileptiform discharges (Hauf et al., 2012). Whilst the analysis of fMRI data is most frequently univariate, i.e., by paired categorical analysis using statistical parametric mapping, recent attempts have shifted toward understanding how multiple brain regions interact with one another. From a theoretical point of view, distributed networks are obscured by categorical analysis because subtraction methods are univariate, i.e., image voxels are analyzed independently. Categorical analysis thus has several limitations. It may overlook parts of a network that do not attain the defined level of significance, or vice versa, may resemble activations incidental to the studied phenomenon. Covariance analysis, in contrast, determines voxels of the brain that exhibit BOLD signal fluctuations correlated in time at low frequencies (≲ 0.1 Hz). This type of functional connectivity resembles networks of brain areas that reveal synchronized neural activity among topographically distinct regions.

Recently, a set of 23 independent networks has been identified in a sample of 180 healthy subjects (Doucet et al., 2011). They correspond to the so-called intrinsic and extrinsic systems, which are associated with internal- and external-oriented processing, respectively. The most frequently reported intrinsic module is the default mode network (DMN). These brain areas are typically active during rest and deactivated during tasks requiring attention such as visuo-spatial tasks (Greicius et al., 2003). The extrinsic modules include parietal the sensori-motor network (SMN), the frontal attention network (FAN), the visual (VIN), and auditory networks (AUN) as well as the working memory network (WMN).

The analysis of covariance in the BOLD signal is nowadays most frequently performed by independent component analysis (ICA). While region of interest (ROI) based approaches have focused on *a priori* assumptions, i.e., the presence of functional connectivity is assumed from previous hypotheses, data-driven approaches as ICA offer the advantage to analyze coherent physiological signals on the whole brain level. Several implementations, most frequently based on the FastICA algorithm (Hyvärinen and Oja, 1997; Hyvärinen, 1999), have been provided to disentangle mixed signals into mutually least dependent spatial source signals that represent different networks following a similar temporal pattern. A frequent assumption is that *N*^src^ spatial “sources” **s** are linearly mixed by a constant *N*^obs^ × *N*^src^ matrix **A** to yield the *N*^obs^ “observations” **x** in the following way:

(1) x=A⋅s

both, **A** and **s** are *a priori* unknown. Here and in the sequel **s** and **x** are matrix notations for *s_li_* with *l* = 1, …, *N*^src^ and *x_ti_* with *t* = 1, …, *N*^obs^, respectively. The index *i* = 1, …, *N*^spc^ with *N*^spc^ ≫ *N*^src^, *N*^obs^ numbers the spatial degrees of freedom and will be omitted from now on to ease the notation. In ICA and related techniques the mixing matrix **A** is estimated by the requirement that the *s_l_* become as independent as possible.

In spatial single-subject ICA the columns of the “mixing matrix” **A** of equation (1) represent the time courses of the independent components (IC) and the matrix elements *A_tl_* inform how strongly and with which sign the source *s_l_* contributes to the observation *x_t_*. At a group level different approaches to ICA have been developed, either performing a secondary analysis on preselected single-subject ICs or methods in which raw single-subject data is integrated before analysis (for reviews, see Guo and Pagnoni, 2008; Calhoun et al., 2009). This includes group ICA approaches in which single-subject data is concatenated in time (Calhoun et al., 2001; Beckmann et al., 2005; Schöpf et al., 2010b) or space (Svensén et al., 2002; Schmithorst and Holland, 2004) or by using a three-dimensional tensor representing spatial, temporal, and subject-specific loadings for each group component (Beckmann and Smith, 2005). Group ICA methods after single-subject ICA have been introduced by selecting the single-subject ICs by visual inspection (Harrison et al., 2008), based on a spatial template (Calhoun et al., 2008), or based on the spatial correlation of the single-subject maps (Esposito et al., 2005; De Luca et al., 2006; Schöpf et al., 2010a, 2011; Varoquaux et al., 2010). A different technique that deserves mentioning in this context is “IC dictionary” creation using “bagged clustering” over a large number of single-subject ICs (Anderson et al., 2011). This approach first reduces dimensionality by projection onto anatomical ROIs and subsequently pools the data by *k*-means clustering.

By construction the ICs of fMRI data are not necessarily related to the BOLD effect. Rather, all kinds of physiological or non-physiological artifacts may appear in ICs. As their removal reduces the noise level in the data, several attempts to automated artifact classification of ICs have been undertaken. In McKeown (2000) a hybrid approach was proposed that combined data-driven spatial ICA with task-related a priory hypotheses that could be analyzed by the general linear model (GLM). IC maps explained by task-related head motion were identified in Kochiyama et al. (2005) by statistically examining task-related intensity and variance changes of the BOLD signals. Both methods require the presence of tasks to enable classification. In contrast, the method proposed by Thomas et al. (2002) used the power spectrum of IC time courses to classify them as candidates for white or structured noise (physiological fluctuations). Perlbarg et al. (2007) used manually defined regions of interest (ROIs) to define typical time courses of structured noise in fMRI data, which were used as regressors for the BOLD signals.

Also spatial features have been employed for artifact identification. A combination of six temporal and spatial features was used in Tohka et al. (2008) to classify ICs from fMRI data in event related and block design. Motivated by typical “IC fingerprints” (De Martino et al., 2007) in Sui et al. (2009) spatial correlation with tissue class templates as well as spatial structure and information content was used to identify artifactual IC maps.

So far, most attempts to automated IC classification were either designed for task/event related fMRI data or rely on spatial information. To our knowledge, automated time course based artifact identification suitable for resting-state fMRI data has not yet been undertaken. In the present contribution we propose a conceptually simple algorithm for unsupervised identification (and potentially removal) of artifactual single-subject ICs, which is entirely based on the time courses. After training on six datasets the algorithm is tested in 29 data sets and classification accuracy is compared to visual rating. Thereafter, the filtered data is subjected to a secondary ICA analysis to illustrate the impact of artifactual ICs on group studies.

## 2 Materials and Methods

### 2.1 Subjects and data acquisition

The data used in the present study consisted of 35 subjects that participated as healthy volunteers in a multiple sclerosis study. The study was approved by the ethics commission of the Canton of Bern. Demographics were chosen to match those of multiple sclerosis patients presenting at the neurological outpatient clinic of the Inselspital in Bern, see Table 1.

**Table 1:** **Demography of subject groups**.

		Training set	Test set	Difference between sets
		*N*^subj^ = 6 *N*^obs^ = 270	*N*^subj^ = 29 *N*^obs^ = 300	
Age (years)	Range	26–42	21–61	*p_U_* = 0.69
	M	32.3	35.3	
	SD	6.7	11.0	
Gender	Male/female	1/5	8/21	*p*_χ_ = 0.72
Handedness	Right/ambidexter/left	6/0/0	27/2/0	*p*_χ_ = 0.36

*Test for equal median age: Mann-Whitney-Wilcoxon *U*-test. Test for equal gender and handedness distribution: *χ*^2^-test with one (gender) and two (handedness) degrees of freedom*.

All subjects underwent T2*-weighted functional and T1-weighted high resolution structural MR imaging. Imaging was performed at the University Institute of Diagnostic and Interventional Neuroradiology, Inselspital, Bern (Rajeev Kumar Verma) on a 3-T Siemens Scanner (Magnetom Verio^®^, Siemens Medical Solutions, Erlangen, Germany) using a 32-channel head coil. Head motion was minimized by fitting foam pads between head and coil. Scanner noise was reduced by using ear plugs.

Resting-state functional images were acquired with a standard EPI sequence and analyzed in detail. In two groups BOLD data were registered with the same MR parameters: repetition time (TR) 1980 ms; echo time (TE) 30 ms; flip angle 90°; inversion time(TI) 910 ms; slice thickness 4 mm; field of view (FOV) 192 mm (matrix size 192 × 192); voxel size 3.0 mm × 3.0 mm × 4.0 mm. The “training data set” and “test data set” consisted of *N*^subj^ = 6 and *N*^subj^ = 29 subjects, respectively, where *N*^obs^ = 270 and *N*^obs^ = 300 volumes were registered. The shorter data sets were acquired earlier than the longer ones, i.e., the groups are not randomized. Notwithstanding, age, gender, and handedness distributions were not significantly different between the groups, see Table 1.

For anatomical co-registration three-dimensional T1-weighted images were obtained using the Modified Driven Equilibrium Fourier Transformation (MDEFT) sequence. The acquisition was performed with the following parameters: TR = 7.92 ms; TE = 2.48 ms; flip angle = 16°; slices per slab = 176; slice thickness 1 mm; FOV = 256 mm (matrix size = 256 × 256), with a resulting voxel size of 1.0 mm × 1.0 mm × 1.0 mm.

### 2.2 Data pre-processing

Pre-processing and analysis of resting-state fMRI data was performed independently for each subject using the freely available FMRIB’s Software Library FSL (http://www.fmrib.ox.ac.uk/fsl/), version 4.1.7. Analysis was done on a Quadcore computer with Intel Xenon^®^ CPU at 2.4 GHz and 12 GB memory under the 64-bit version of Ubuntu Linux 12.04 LTS.

The pre-processing stream was as follows: Motion correction was carried out using the MCFLIRT tool and slice timing was corrected. The BET tool was used for brain extraction in structural and functional MR data and spatial smoothing with a 6-mm FWHM kernel was performed for functional data. The time constant for the high pass filter was set to 111 s, leaving only frequencies *f* > 0.009 Hz in the pre-processed BOLD time course.

fMRI data were first registered to each subject’s high resolution structural images (MDEFT). Subsequently, the BOLD data were registered to the standard MNI space. For both registration steps linear transformations with 12 degrees of freedom (translation, rotation, scaling, sheering) were used.

### 2.3 Single-subject ICA

Least dependent components in the BOLD maps were estimated for each subject separately. The number of single-subject sources $N_{n}^{\text{src}}$ was estimated from the data for each subject by maximizing the Laplacian estimate to the Bayesian evidence of the model order (Minka, 2000; Beckmann and Smith, 2004). After dimensionality reduction by principal component analysis (PCA) single-subject ICA was performed using probabilistic ICA (Beckmann and Smith, 2004) as implemented in version 3.10 of FSL’s MELODIC toolbox.

#### 2.3.1 Supervised post-processing

The MELODIC output includes a collection of spatial maps, some of which represent physiological RSNs and some of which represent artifacts. For visual artifact identification the following criteria were applied by three raters independently (Christian Rummel, Eugenio Abela, and José Fernando Zapata Berruecos), both in the training as well as in the test data set. Maps were marked as obvious artifacts if the activations were confined:

(a) to the boundaries of the brain,
(b) to the cerebral ventricles,
(c) to the inter-hemispheric scission, or
(d) to less than three slices.

In addition, maps were marked as artifacts:

(e) if the activations were distributed irregularly over the whole parenchyma without clear regions of accumulation,
(f) if the time course resembled one or several motion correction parameters, or
(g) if the power spectrum of the time course was extraordinarily broad or narrow.

After independent rating the raters agreed on obvious artifact ICs and potential RSNs in a discussion session. The rating sensitivities:

(2) $${\text{sens}}_{n}=\frac{{\text{TP}}_{n}}{{\text{TP}}_{n}+{\text{FN}}_{n}}\;,$$

specificities

(3) $${\text{spec}}_{n}=\frac{{\text{TN}}_{n}}{{\text{FP}}_{n}+{\text{TN}}_{n}}$$

and accuracies

(4) $${\text{acc}}_{n}=\frac{{\text{TP}}_{n}+{\text{TN}}_{n}}{{\text{TP}}_{n}+{\text{FN}}_{n}+{\text{FP}}_{n}+{\text{TN}}_{n}}=\frac{{\text{TP}}_{n}+{\text{TN}}_{n}}{N_{n}^{\text{src}}}$$

were calculated subject-wise. In equations (2–4) TP*_n_* and TN*_n_* denote the numbers of true positives and true negatives in subject *n* = 1, …, *N*^subj^ (i.e., the number of single-subject ICs rated the same way by an individual rater and in the agreement of all raters). Similarly, FP*_n_* and FN*_n_* are the numbers of false positives and false negatives (i.e., the number of single-subject ICs with disagreement).

#### 2.3.2 Automated post-processing

The problem of automatic classification of ICs has been approached in De Martino et al. (2007), Tohka et al. (2008) by subjecting multi-dimensional feature vectors to support vector machines or global decision trees, respectively. Here, we do not aim at full IC classification. Rather, our objective is automated identification of single-subject ICs that are obviously artifacts. To this end we implemented two simple time course based criteria in an automatic filtering process:

(I) A GLM was fitted to the time course *s_l_* of each single-subject IC with the motion correction parameters as regressors (three translations, three rotations). If the significance *p*_moco_ of Pearson’s correlation coefficient between *s_l_* and the GLM prediction was smaller than a threshold $p_{\text{moco}}^{\text{crit}}\in \left[0,1\right]$ the component was discarded as probable artifact of residual subject motion.
(II) The spectral power density of the IC time courses *s_l_* was estimated and filtered in the frequency band 0.009 < *f* < 0.08 Hz, where RSN associated spontaneous BOLD fluctuations are expected (Biswal et al., 1995; Weissenbacher et al., 2009; Schöpf et al., 2010a). If the null hypothesis that the original and the filtered power distribution are compatible was rejected on a significance threshold $p_{\text{pow}}^{\text{crit}}\in \left[0,1\right]$ by a Kolmogorov-Smirnov test (Siegel, 1956) the corresponding components were also interpreted as artifacts.

The criteria (I) and (II) of the automated filter represent a quantitative formulation of the time course based visual criteria (f) and (g) above. No information about the spatial distribution of activations was used.

Both thresholds $p_{\text{moco}}^{\text{crit}}$ and $p_{\text{pow}}^{\text{crit}}$ were chosen in a data driven way by optimizing the agreement between automated and visual analysis of single-subject IC maps in the training set. To this end the parameter space was systematically scanned in 10^−50^ ≤ *p*_moco_, *p*_pow_ ≤ 10^−1^ and subject-wise agreement between automatic and visual rating (agreement of all raters) was assessed by the accuracy of the discriminator as defined in equation (4). The mean 〈acc*_n_*〉 over the single-subject accuracies of the filter defined in equation (4) was maximized. As opposed to maximization of the global accuracy:

(5) $${\text{acc}}_{\text{glob}}=\frac{\sum_{n}\;{\text{TP}}_{n}+\sum_{n}\;{\text{TN}}_{n}}{\sum_{n}\;N_{n}^{\text{src}}}$$

of all single-subject ICs from all subjects this prevents over-tuning the parameters for training subjects with large $N_{n}^{\text{src}}.$ The same thresholds $p_{\text{moco}}^{\text{crit}}$ and $p_{\text{pow}}^{\text{crit}}$ were subsequently used in the test set.

### 2.4 Simple approach to group ICA

Group ICA was performed by concatenating single-subject IC maps from all *N*^subj^ subjects in time and performing a secondary ICA on the joint data set using MELODIC. As in the single-subject case we estimated the number of group ICs using the Laplacian approximation to the model order (Minka, 2000; Beckmann and Smith, 2004). Comparing results for filtered and unfiltered single-subject ICs we briefly illustrate the potential impact of artifactual ICs on group ICA studies.

### 2.5 Statistical evaluation

Statistical analysis was performed using Matlab 7.0.4 (MathWorks, Natick, MA, USA). As for our group sizes most often at least one distribution is small (*N* < 10) significance of different medians between *k* distributions was tested non-parametrically by the Mann-Whitney-Wilcoxon *U*-test for *k* = 2 and by the Kruskal-Wallis test for *k* > 2 (Siegel, 1956). Difference in discrete distributions was tested by the *χ*^2^-test (Siegel, 1956). As significance level we chose α = 0.01 for all tests. Results were interpreted as “marginally significant” or “trends” if *p* < 0.05.

## 3 Results

### 3.1 Single-subject ICA

**Figure 1:**
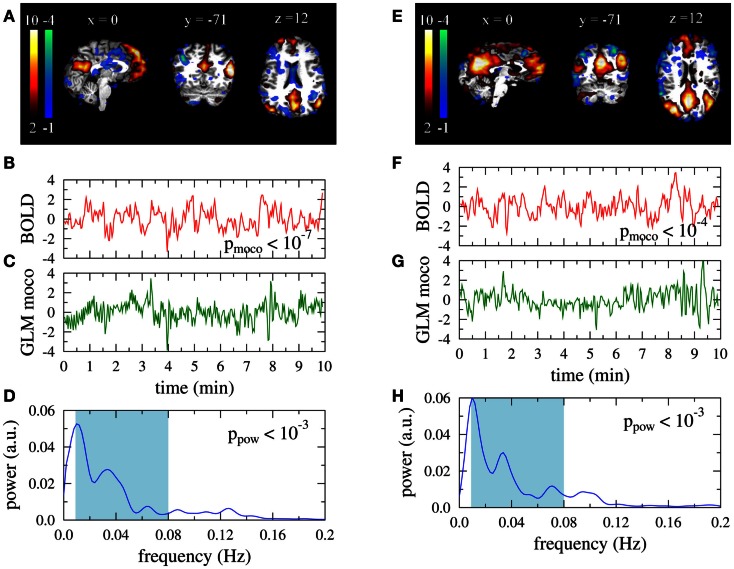
**Default mode networks for two subjects**. Left: 37 year old male, right: 35 year old female participant. **(A,E)** Activation maps. Colorbars represent z-scores of IC weights per voxel. Data are presented in native space. **(B,F)** Associated BOLD time courses (normalized to zero mean and unit variance). **(C,G)** Best fit of a GLM with motion correction parameters as regressors to the BOLD data. **(D,H)** Power spectra of the BOLD time courses.

In Figure 1 we illustrate the results for the DMN exemplarily in two subjects. Figures 1A,E give an overview of the typical between-subject variability of the representation of the functional IC maps on the individual anatomies. The corresponding time courses *s_l_* are displayed in Figures 1B,F and the best fit of a GLM with the six motion correction parameters as regressors in Figures 1C,G. Correlation is significant in both subjects. Figures 1D,H show the power spectra of the DMN time courses. Filtering in the range 0.009 < *f* < 0.08 Hz (shaded in light blue) leads to significant changes of the power distribution.

**Figure 2:**
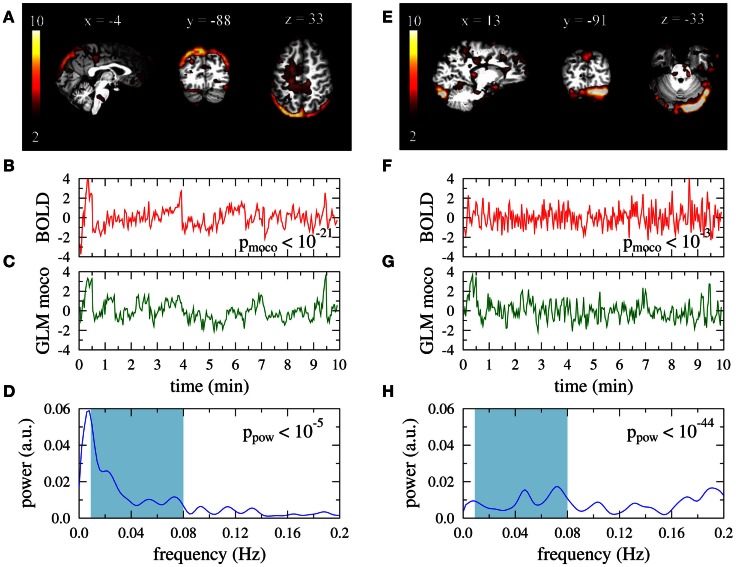
**Artifact related single-subject ICs that are excluded by the automatic filter**. Data is taken from a 35-year old female participant. Right: typical type I artifact (residual subject motion), left: typical type II artifact (too much power in high frequencies). **(A,E)** Activation maps. Colorbars represent z-scores of IC weights per voxel. Data are presented in native space. **(B,F)** Associated BOLD time courses (normalized to zero mean and unit variance). **(C,G)** Best fit of a GLM with motion correction parameters as regressors to the BOLD data. **(D,H)** Power spectra of the BOLD time courses.

Two obviously artifactual ICs are shown in Figure 2. Data was taken from the same subject as the right column in Figure 1. The IC of the left column is clearly related to residual subject motion, leading to a much smaller value *p*_moco_ than observed in Figure 1B. The activations of the IC map were mainly confined to the brain boundaries (Figure 1A). Other examples of this artifact type are characterized by typical activation “halos” in axial slices. In contrast to *p*_moco_ the power related *p*_pow_ is in the same range as for the DMNs in Figure 1. A typical power related artifact IC is displayed in the right column. Correlation between the time course and the GLM of the motion correction parameters is the same as in Figure 1. However, the time course has much more power in large frequencies (see Figures 1F,H), leading to a much smaller value of *p*_pow_. In contrast to the motion related artifact here the IC map is much less suspicious on its own (Figure 1E). However, the asymmetry and the strong involvement of the cerebellum confirm this IC as artifact in visual inspection.

**Figure 3:**
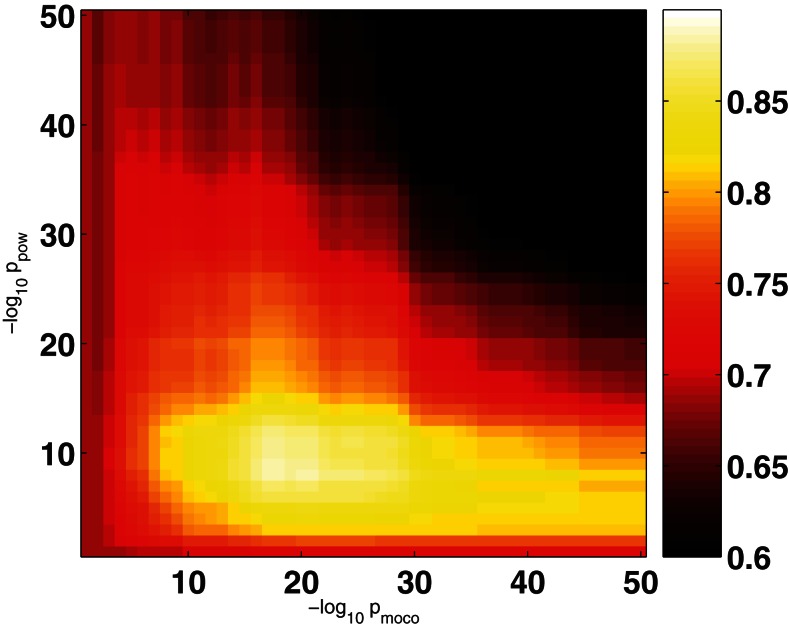
**Average filter accuracy in the training set as a function of −log_10_ (*p*_moco_) and −log_10_ (*p*_pow_)**.

The large separation of *p*-values between obviously artifact related ICs and ICs that might represent RSNs allowed the construction of the proposed time course based automated artifact filter. In Figure 3 the objective function 〈acc*_n_*〉 is displayed for the training set as a function of the two thresholds *p*_moco_ and *p*_pow_ (logarithmic scale). The mean of the accuracies defined in equation (4) is maximized by the choice $p_{\text{moco}}^{\text{crit}}=10^{-17}$ and $p_{\text{pow}}^{\text{crit}}=10^{-8}$, where 〈acc*_n_*〉 = 0.88. Note that in the range 10^−25^ < *p*_moco_ < 10^−15^ and 10^−15^ < *p*_pow_ < 10^−5^ the mean accuracy is rather insensitive to the precise choice of the thresholds.

In Table 2 the number of single-subject ICs are compiled. Neither the MELODIC estimate $N_{n}^{\text{src}}$ nor the number of potential RSNs that passed the visual rating or the automatic filtering process (i.e., ICs that were not automatically rated as obvious artifacts) were significantly different between the training and the test data set. Starting from the Laplacian estimator for $N_{n}^{\text{src}},$ approximately 2/3 of the single-subject ICs were concordantly rated as obvious artifacts in both groups by the raters and the filter.

**Table 2:** **Number of single-subject ICs as proposed by MELODIC and number of potential RSNs (i.e., ICs that were not rated as obvious artifacts by visual inspection or the automated filter)**.

		Training set	Test set	Difference between sets
$N_{n}^{\text{src}}$	Range	35–48	29–84	*p_U_* = 0.57
	M	42.3	45.4	
	SD	6.0	9.1	
$N_{n}^{\text{RSN}}$ (visual insp.)	Range	11–17	3–30	*p_U_* = 0.77
	M	13.2	13.9	
	SD	2.1	4.7	
$N_{n}^{\text{RSN}}$ (filter)	Range	8–24	5–26	*p_U_* = 0.28
	M	13.3	15.6	
	SD	5.8	5.5	
Difference visual vs. filter		*p_U_* = 0.78	*p_U_* = 0.18	

*Test for equal medians: Mann-Whitney-Wilcoxon *U*-test*.

Neither the visual rating accuracies (i.e., single rater opinion as compared to inter-rater agreement) nor the filter accuracies were significantly different between the data sets, see Table 3. However, the smallest obtained accuracies were much smaller in the test set than in the training set, especially for the filter. Although the overall accuracy of the proposed time course based filter was rather high (mean accuracy 0.80 in out-of-sample tests) it did not reach the performance of human raters. The difference was much more significant in the test set than in the training set. Rating sensitivities and specificities are compiled in Tables 4 and 5. The only difference between the data sets was a trend toward smaller specificity of the automated filter in the test set (*p_U_* = 0.03). While in the training data set sensitivity and specificity of the filter were only marginally smaller than for human raters the differences were significant in the test set.

**Table 3:** **Rating accuracies of individual raters and automated filter as compared to the raters’ agreement**.

		Training set	Test set	Difference between sets
acc*_n_* (visual insp.)	Range	0.95–1.00	0.82–1.00	*p_U_* = 0.23
	M	0.98	0.96	
	SD	0.02	0.04	
acc*_n_* (filter)	Range	0.76–0.94	0.41–0.96	*p_U_* = 0.12
	M	0.88	0.80	
	SD	0.07	0.11	
Difference visual vs. filter		*p_U_* < 10^−3^	*p_U_* < 10^−11^	

*Test for equal medians: Mann-Whitney-Wilcoxon *U*-test*.

**Table 4:** **Rating sensitivities of individual raters and automated filter as compared to the raters’ agreement**.

		Training set	Test set	Difference between sets
sens*_n_* (visual insp.)	Range	0.94–1.00	0.83–1.00	*p_U_* = 0.79
	M	0.97	0.97	
	SD	0.02	0.04	
sens*_n_* (filter)	Range	0.62–1.00	0.58–1.00	*p_U_* = 0.17
	M	0.89	0.82	
	SD	0.14	0.11	
Difference visual vs. filter		*p_U_* = 0.03	*p_U_* < 10^−10^	

*Test for equal medians: Mann-Whitney-Wilcoxon *U*-test*.

**Table 5:** **Rating specificities of individual raters and automated filter as compared to the raters’ agreement**.

		Training set	Test set	Difference between sets
spec*_n_* (visual insp.)	Range	0.92–1.00	0.67–1.00	*p_U_* = 0.81
	M	0.97	0.94	
	SD	0.04	0.09	
spec*_n_* (filter)	Range	0.67–1.00	0.10–1.00	*p_U_* = 0.03
	M	0.81	0.75	
	SD	0.14	0.23	
Difference visual vs. filter		*p_U_* = 0.02	*p_U_* < 10^−6^	

*Test for equal medians: Mann-Whitney-Wilcoxon *U*-test*.

### 3.2 Group ICA

**Figure 4:**
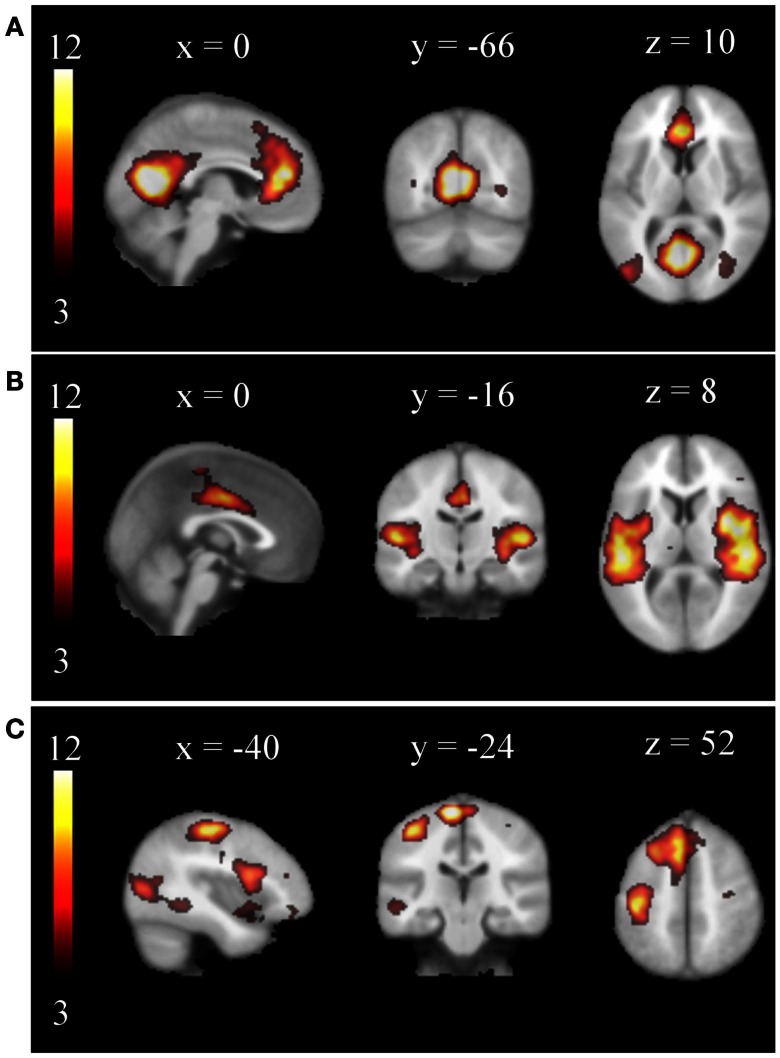
**Examples of group RSNs obtained from the test data set by a secondary ICA procedure: (A) DMN, (B) AUN, (C) WMN**. Obvious artifacts were automatically removed before concatenation of the single-subject maps. The networks are displayed on a standard brain in MNI space.

Concatenating all single-subject IC maps from all subjects in time (254 in the training set and 1356 in the test set) and performing a secondary ICA the MELODIC toolbox respectively estimated 29 and 581 group ICs (Laplacian method). Especially the number obtained for the test data set is of course much too large. In consequence, the vast majority of obtained group ICs were obviously artifactual and none of the typical RSNs was obtained. Rather, some ICs seemed to resemble fragments of known RSNs. After automated removal of the artifact ICs by the proposed filter, the secondary ICA revealed 14 and 59 group ICs in the training and test data sets, respectively. Many of the established RSNs were found as, e.g., the DMN, the SMN, the AUN, the VIN, and the WMN. Examples are compiled in Figure 4.

## 4 Discussion

In this contribution we proposed a simple filter for automated identification of obviously artifactual single-subject ICs. The filter relies on only two features of the associated IC time courses: (I) correlation with motion correction parameters and (II) power outside the expected range 0.009 < *f* < 0.08 Hz. Thresholds were deduced from a training data set of six subjects. The maximum of the mean subject-wise in-sample accuracy was found unique and broad (Figure 3) and thus smaller variations of the threshold parameters are not expected to influence our results sizably. In addition, alternative implementations of the rules (I) and (II) are conceivable. For example, the *p*-value of the KS-test for the original and filtered power spectra could be replaced by a criterion on the spectral width or a threshold for the fraction of power found outside the allowed region.

The filter was applied to an out-of-sample test data set of 29 subjects. Neither the group demographics (see Table 1) nor the rater and filter performance were significantly different between the training and test data set (see Tables 3– 5). We take these findings as a confirmation that the non-random selection of training and test data did not induce a bias.

Although our results for the test data set indicate that the automated artifact filter does not reach the performance of visual inspection by human raters, we consider the mean out-of-sample accuracy of 0.80 (mean sensitivity 0.82, mean specificity 0.75) high enough to considerably aid or replace user intervention in large data sets. As expected, performance differences between human raters and the filter were much more significant in the test than in the training data set. Besides the fact that in-sample performance is optimized whereas out-of-sample performance is not, the better statistics due to five times larger number of subjects may be the main explanation for this finding. As filters will almost always be trained on limited data and applied to larger sets, we consider this a realistic setting.

In contrast to the proposal by Sui et al. (2009), where only spatial information of IC maps was used, our artifact detector is entirely based on properties of the IC related BOLD time courses. However, as can be seen in Figures 2A,E, these are reflected by suspicious visual appearance of the IC maps. This suggests that map based identification of head movement related artifacts affecting mainly the brain boundaries (Tohka et al., 2008) could probably be replaced by our conceptually simpler criteria. Inclusion of our criteria (I) and (II) in a combination of non-related temporal and spatial features similar to the classification approaches by De Martino et al. (2007), Tohka et al. (2008) could possibly help to improve filter performances considerably.

Our rule (II) has similarity with the power spectrum based classification into structured or white noise time courses in Thomas et al. (2002) and the two signal power dependent features of Tohka et al. (2008). The criterion (I) is related to the methods by McKeown (2000), Kochiyama et al. (2005). However, these methods rely on the presence of tasks and are consequently not applicable to resting-state fMRI. In contrast, our proposal of using motion correction parameters in a GLM may also be suitable to distinguish task-related activations from task-related movement artifacts. The approach by Perlbarg et al. (2007) uses physiological noise time courses as regressors. Here, an important difference is that our proposal does not require manual user intervention for ROI definition.

Also the recent publication by Kundu et al. (2012) deserves discussion. Measuring at three echo times (TE) a differentiation between BOLD and non-BOLD signals in fMRI data was possible. However, this method requires acquisition of multi-echo echo planar imaging (EPI) sequences and can of course not be applied retrospectively to standard EPI data.

To illustrate the impact of artifact ICs on group studies we used a secondary ICA on top of full and artifact corrected single-subject ICA output. Considerable improvement was found in the sense that typical RSNs were obtained only after exclusion of artifacts. We used a simple group analysis strategy, which is similar to the approach implemented in GIFT (Calhoun et al., 2001, 2009). Spatial maps from all *N*^subj^ subjects are processed jointly by an arbitrary ICA algorithm. An important difference is that in our approach the number $N_{n}^{\text{src}}$ of single-subject ICs is estimated individually for each subject *n* = 1, …, *N*^subj^, while in GIFT a PCA based dimensionality reduction is performed to the *same* predefined number $N_{\text{fix}}^{\text{src}}$ in all subjects. This bears the risk of subjecting noise ICs to the second level analysis in some subjects, while potentially eliminating ICs of interest in others. A common advantage of GIFT and the secondary ICA procedure is that the respective data dimensions $N^{\left(2\right)}=N^{\text{subj}}\cdot N_{\text{fix}}^{\text{src}}$ and $N^{\left(2\right)}=\sum_{n}\;N_{n}^{\text{src}}$ are usually much smaller than for straight forward temporal concatenation, where $N^{\left(2\right)}=N^{\text{subj}}\cdot N^{\text{obs}}.$

## Conflict of Interest Statement

The authors declare that the research was conducted in the absence of any commercial or financial relationships that could be construed as a potential conflict of interest.
